# Interprofessional Quality Improvement Project to Reduce the Length of Stay of Tracheostomized Patients in a Multi-Etiological Intensive Care Unit—The Contribution of Speech and Language Therapy to the Overall Result (IQ-ICU-SLT)

**DOI:** 10.3390/jcm15010303

**Published:** 2025-12-31

**Authors:** Jürgen Konradi, Isabella Neef, Lukas Müller, Robert Kuchen, Heike Maagh, Ulrich Betz, Marc Bodenstein

**Affiliations:** 1 Institute of Physical Therapy, Prevention and Rehabilitation, University Medical Center of the Johannes Gutenberg University Mainz, 55131 Mainz, Germany; lukaswerner.mueller@unimedizin-mainz.de (L.M.); heike.maagh@unimedizin-mainz.de (H.M.); ulrich.betz@unimedizin-mainz.de (U.B.); 2 Department of Anesthesiology, University Medical Center of the Johannes Gutenberg University Mainz, 55131 Mainz, Germany; ineef@students.uni-mainz.de (I.N.); marc.bodenstein@unimedizin-mainz.de (M.B.); 3 Institute of Medical Biostatistics, Epidemiology and Informatics, University Medical Center of the Johannes Gutenberg University Mainz, 55131 Mainz, Germany; robert.kuchen@uni-mainz.de

**Keywords:** tracheostomy, decannulation, ICU, LOS, SLT, quality management

## Abstract

**Background/Objectives:** Reasons for long-term stays in intensive care units (ICUs) include various critical conditions, prolonged weaning with post-extubation dysphagia (PED), as well as the mere presence of a tracheal cannula. In an interprofessional QM project, medicine, nursing, physiotherapy, speech and language therapy (SLT), and occupational therapy work together to reduce the length of stay (LOS) in ICUs. SLT focuses on tracheal cannula management (TCM) and PED. The primary aim of SLT is fast and safe decannulation and thereby the reduction in LOS. **Methods:** Two SOPs for dealing with PED patients and for structured TCM were developed for this purpose and were both implemented in a postoperative ICU, together with a SLT staff increase. To compare the effects on the intervention group (IG, *n* = 54), a historical control (HC, *n* = 58) group was created through a retrospective data analysis. We screened all patients from ICU (*n* = 5605), including those with tracheostomy, and analyzed them during their ICU stay. **Results:** Clinically relevant results were observed for the mean time in days of tracheostomy in those who could be decannulated (HC = 43.43, IG = 23.8; d = 0.99) and, even more importantly, for LOS in days (HC = 33.41, IG = 23.8; d = 0.48). **Conclusions:** The integration of SLT in ICU care is feasible and helps to reduce the time to decannulation and LOS.

## 1. Introduction

Patients who remain in intensive care units (ICUs) for extended periods face a high disease burden and increased risks of illness and death. Factors contributing to extended ICU stays include multiple organ failure, unstable circulation, delirium, prolonged ventilator weaning with post-extubation dysphagia (PED), and the presence of a tracheal cannula (TC), which can complicate transfers to other wards or care facilities due to their limited capacity to manage such cases [1]. Tracheostomized ICU patients experience an even greater burden, with longer hospital and ICU stays, higher mortality rates, and a greater chance of being discharged to a care facility. These patients often have severe underlying conditions, and tracheostomy may prompt a reassessment of patient goals and advanced care planning [2]. Strong respiratory function, demonstrated by powerful cough reflexes and efficient secretion control, is reported as a fundamental predictive factor for successful decannulation [3]. Ideally, decannulation should occur during or at the conclusion of the patient’s stay in the ICU [4] to minimize the occurrence of further complications.

A recent national anesthesiology guideline recommends multiprofessional team efforts in order to improve the quality of treatment and shorten the LOS. However, the guideline focuses on the effects of mobilization and thereby on the physiotherapeutic role in the treatment process [5]. A national [6] and an international [7] guideline of intensive care societies both recommend broad staff education and strongly recommend to make speech and language therapy (SLT) available for ICU up to the point that “all patients with a tracheostomy must have communication and swallowing needs assessed by an SLT” [7]. Especially, SLT and there competencies in tracheostomy and dysphagia management [8] could provide additional input for intensive care treatment quality beyond their ancestral field of neurologic rehabilitation [9] and have already demonstrated positive effects on aspects such as pneumonia prevention and length of stay (LOS) in quality management (QM) projects [10,11]. Furthermore, there is evidence that the mere application of structured decannulation pathway is able reduce the total time to decannulation [12].

Therefore, in order to not only capture the best moment to discharge the patient, but also to facilitate the discharge process, we started an interprofessional QM project (IQ-ICU). This project integrates the work of medicine, nursing, physiotherapy, SLT, and occupational therapy to improve the quality of treatment, reduce morbidity, and reduce the length of stay (LOS) in the ICU, with each profession providing their respective [8]. SLT focuses primarily on the aspects of tracheal cannula management and dysphagia. In order to evaluate the effect of IQ-ICU, we retrospectively analyzed patient data one year prior to the implementation phase and compared this historic control group (HC) to an intervention group (IG) one year after the full start of the implemented QM project.

In this paper we will focus on the SLT part of the whole IQ project and its effect on endpoints associated with tracheostomy and dysphagia (IQ-ICU-SLT). The primary aim of the SLT was the fastest possible decannulation with maximum patient safety, and with that ideally a reduction in LOS on ICU.

## 2. Materials and Methods

Before the start of the IQ-ICU project, there were no specific SOPs dealing with dysphagia or tracheal cannula management for intensive care medicine. Decisions regarding oral feeding or decannulation were based on clinical experience mostly of the nurses and physicians. Only physiotherapy was part of the daily routine. SLT was only available upon specific requests of the ICU physicians in exceptional cases.

Therefore, we decided to implement a comprehensive concept with specific measurements for SLT treatment, focusing on cannula management and dysphagia, in a postoperative ICU of a German university hospital that operates with up to 43 beds.

### 2.1. Descrition of the Overall IQ-ICU Concept

**Figure 1:**
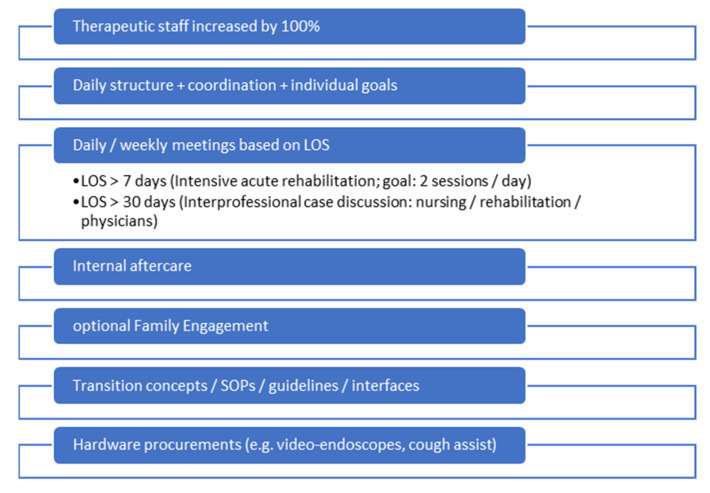
Key points of the interprofessional quality management framework project.

No changes were made regarding the general intensive medical care approach of the physicians. Staffing levels in the therapy area were doubled so that therapy sessions could take place twice a day. An interprofessional Standard Operation Procedure (SOP) for the collaboration of physiotherapy, SLT, and occupational therapy was created. Furthermore, depending on the LOS, a daily interprofessional therapy conference and weekly goal meetings were introduced. The integration of family engagement was executed wherever feasible, and adequate supplies (for example, video endoscopes for flexible evaluation of swallowing and to detect readiness for decannulation) were procured (compare Figure 1 for the key points of the IQ-ICU project). Pharyngeal electrical stimulation (PES; Phagenesis Ltd., UK) was considered, but since the ICU patients do not necessarily meet the necessary etiologies for its application (in Europe, its neurogenic dysphagia), we decided against PES at the first stage of the IQ-ICU project, even though it showed superior results for faster decannulation [13].

### 2.2. Descrition of the SLT Concept

**Figure 2:**
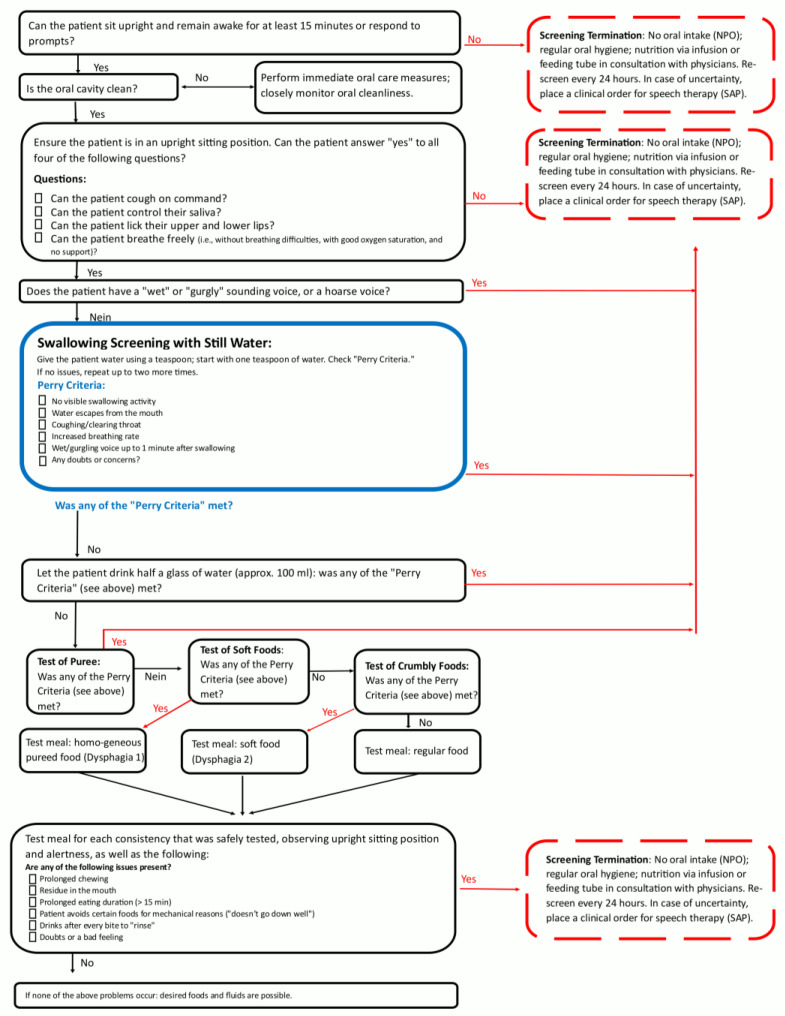
Swallowing screening for the intensive care units and general wards. Pathway for oral intake or clinical examination by Speech and Language Therapist (part of a standard operation procedure for post-extubation dysphagia), derived from Perry et al. (2001) [16].

**Figure 3:**
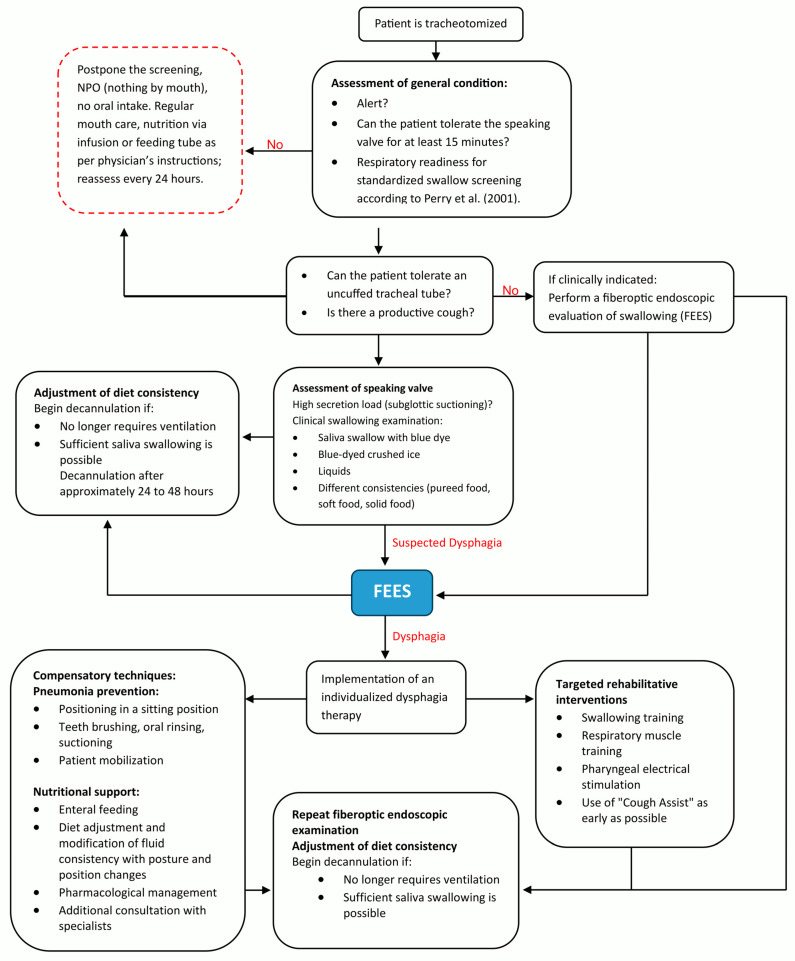
Pathway of the standard operation procedure for structured tracheal cannula management in ICU with special focus on patients with risk of dysphagia based on international expert recommendations of Likar et al. (2024) [17] and Perry et al. (2001) [16].

**Figure 4:**
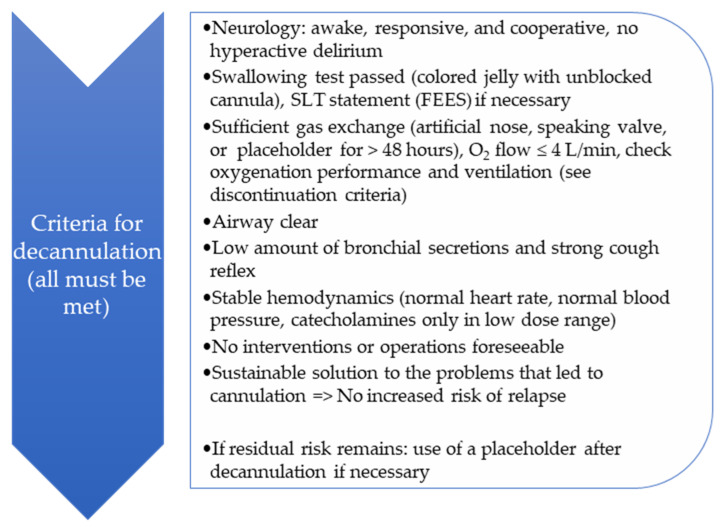
Criteria for decannulation with SLT-specific diagnostic elements that were applied as part of the implementation period between pre and post. The decision to decannulate was made in an interprofessional case discussion.

A relevant part of ICU patient care is the dysphagia and tracheostomy management, with essential contributions from the SLT team. The SLT team consisted of +- seven members across the whole intervention period. Two SOPs (the pathways of the SOPs are depicted in Figure 2 and Figure 3) for dealing with PED patients and for structured tracheal cannula management based on international expert recommendations (3) were developed for this purpose by the SLT team in preparation of the project implementation and were both implemented during in a 3-month period at the beginning of 2024. Every team member was trained for the concept. Additionally, all members are qualified for tracheal cannula management and FEES according to the respective curriculums [14,15] that are accepted by national and international expert societies (German Society for Swallowing Disorders and European Society for Swallowing Disorders). SLT diagnostics comprised either clinical dysphagia examinations and/or FEES. SLT treatment targeted airway and swallowing functions and was based on individualized cannula management (uncuffing, stepwise increase in time with a speaking valve, parameter-based and mutual assessment for decannulation readiness), dysphagia therapy, targeted rehabilitative interventions (e.g., muscle strength training, use of cuff assist), compensatory techniques, and diet adaptations. For a complete overview, see Figure 2 and Figure 3. Beyond the SLT treatment concept, precise criteria for decannulation with SLT-specific diagnostic elements were developed and applied as part of the implementation period between pre and post (see Figure 4). These criterial were used in order to assess readiness for decannulation. All of them had to be met before decannulation was performed. The decision to decannulate was made in an interprofessional case discussion. A further SLT treatment goal was to teach the patients self-care competence for their cannula (i.e., self suctioning). In order to put this into practice, there was a planned SLT staff increase from approximately 0.1 to 0.8 full-time employee equivalents. SLT was allocated to tracheal cannula management if the first assessment of general condition indicated readiness for treatment (see Figure 3).

### 2.3. Study Design, Patient Inclusion and Endpoints

**Figure 5:**
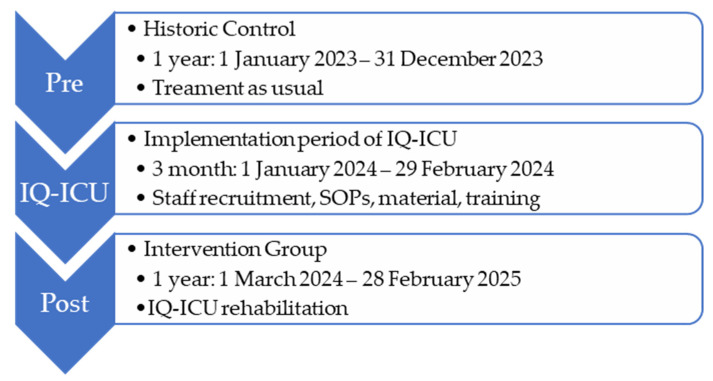
Pre-post study design with project implementation period between pre and post.

As a basis for comparison of the effects on the intervention group (IG), a historical control (HC) group was created through a retrospective data analysis of the year 2023 (Ethics Committee of the Rhineland-Palatinate Chamber of Physicians: 2024-17433). The whole time period of the comparison was across 24 (+3) months in a non-randomized pre-/post study design (see Figure 5). The study is registered with the WHO Register for Clinical Trials (DRKS00036084).

We included all patients who underwent tracheostomy during intensive care stay or later during the hospital stay or who already entered the ICU tracheostomized. No additional exclusion criteria were applied. The primary endpoint of the framework project (IQ-ICU) is a composite endpoint, combining morbidity and the length of stay in ICU. Secondary outcomes were morbidity, amongst many others measured by the Sequential Organ Failure Assessment Score (SOFA, 0–24, organ dysfunction) [18], the Simplified Acute Physiology Score II (SAPS II, 0–163, risk of mortality) [19], the Clinical Frailty Scale (CFS, 0–9, frailty) [20], and functional dependence and independence (Early Rehabilitation Barthel-Index A (−325–0) and B, (0–100) [21], and mortality.

#### SLT-Specific Aspects of Study Design, Patient Inclusion, and Endpoints

As the SLT-specific primary outcome (P1), we considered the group differences in days with tracheal cannula during the ICU stay. Clinically more relevant, but not solely attributable to SLT, is the LOS in the ICU; hence, we considered it as a further primary, but not SLT-specific, outcome (P2). SLT-specific secondary outcomes were the number of possible safe decannulations during the ICU stay, given SLT in minutes, amount of performed clinical and endoscopic swallowing examinations, and competence of self-care for their cannula (i.e., self-suctioning).

This paper specifically analyzes the SLT-related outcomes. In further sub-analyses, the relationship between tracheal cannula management and its potential influence on the LOS will be investigated.

### 2.4. Data Acquisition, Aggregation, and Statistic Analyses

Data were collected from two online patient data management systems by two members of the study group (I.N. and L.M.). All patient data were entered into one pseudonymized Excel sheet table. After several mutual meetings and data curation, it resulted in a clear-to-analyze version. All further statistical calculations (either with Microsoft Excel 2016, Remont, WA, USA, or SPSS v.29, IBM, Armonk, NY, USA) are based on this final version of the raw data table. For inferential statistics of metric variables, we calculated two-sample *t*-tests (two-sided significance) and X^2^-tests for nominal variables. Additionally, for the most relevant outcomes, effect sizes according to Cohen’s D [22] were provided.

## 3. Results

**Figure 6:**
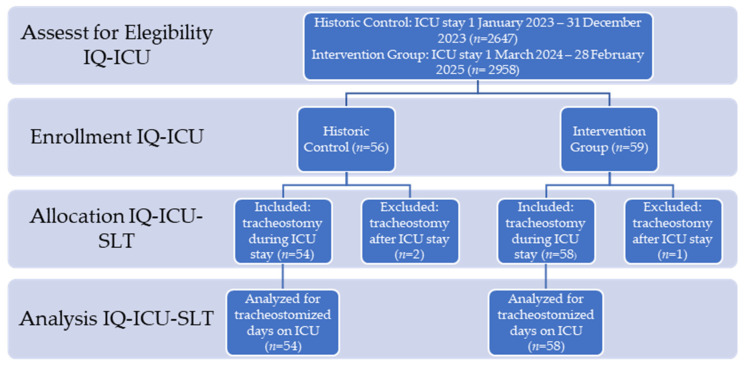
Transparent reporting of trial enrollment and analysis adopted from CONSORT.

After assessment of eligibility of *n* = 5605 patients (HC *n* = 2647; IG *n* = 2958), we enrolled 115 tracheostomized patients during the investigation period who came from a broad-spectrum, postoperative intensive care unit, allocated *n* = 112 (2%) to IQ-ICU-SLT (HC *n* = 54, IG *n* = 58), and analyzed these cases regarding the SLT-specific endpoints (see Figure 6 for details of enrollment).

### 3.1. Patient Characteristics After Enrollment

Amongst the most prominent etiologies were heart-vascular surgery and plastic operations on vessels, surgery of the gastrointestinal tract, and organ replacement therapy.

General patient characteristics across both groups such as the mean age in years (HC = 65.43, IG = 67.75) and BMI (HC = 27.0, IG = 28.06) appear to be comparable, although sex is unequally distributed, with fewer females in the HC (f = 29.6%). Regarding clinical characteristics, both group samples show typical values in ICU-related scores. The risk of morbidity (according to SOFA max) was higher in the HC but not unequally distributed for all other scores. In 20.4% of patients in the HC and in 10.3% of patients in the IG (15.18% across both groups), the complication of apoplexy emerged. Mortality across groups was not unequal. Regarding types of tracheostomies, there were more surgical (primary and secondary) tracheostomies in the IG. This difference also resulted in an earlier performed tracheostomy or first day with a TC in ICU (patients with prior tracheostomy) in the IG (mean of days: HC = 13.93, IG = 9.71) All the most relevant patient and clinical characteristics can be viewed in Table 1.

### 3.2. Results for SLT-Specific Primary and Secondary Study Endpoints

**Figure 7:**
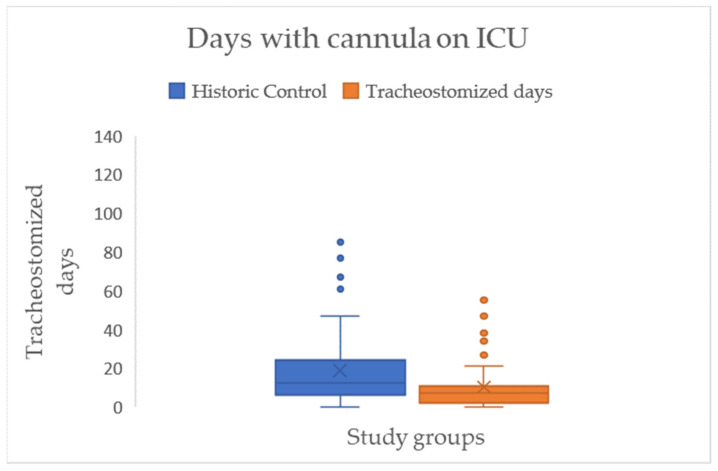
Boxplots with median (-), mean (x), interquartile range (IQR box), whiskers (1.5 × IQR), and outliers (°) for tracheostomized period (days with cannula) during the ICU stay for both study groups.

Across all patients, there is a significant effect for the primary outcome of SLT, resulting in a reduction in the mean number of days with a cannula in the ICU by approximately eight days (HC = 18.85 → IG = 10.31), with a medium effect size (d = 0.54) (see Figure 7/Table 2).

**Figure 8:**
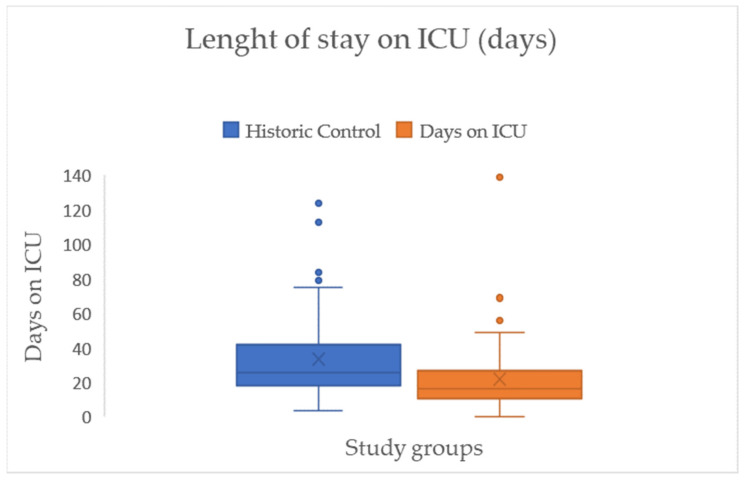
Boxplots with median (-), mean (x), interquartile range (IQR box), whiskers (1.5 × IQR), and outliers (°) for length of stay in ICU in days across both study groups.

This is linked to the LOS in the ICU, which shows a comparable effect size (d = 0.48) and, on average, a significant reduction in mean days between the two groups by approximately twelve days (HC = 33.74 → IG = 21.94) (see Figure 8/Table 2).

**Figure 9:**
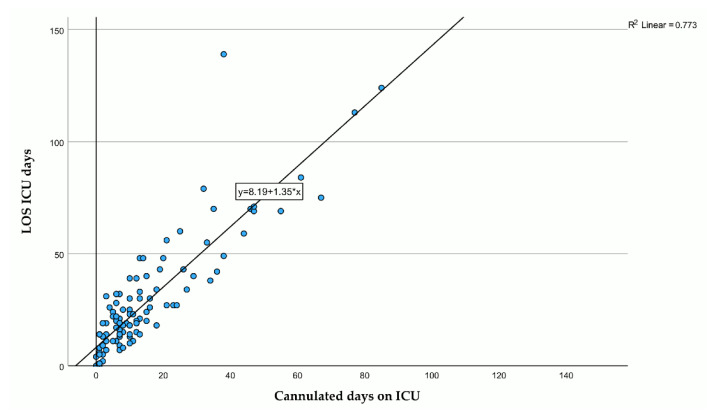
Scatterplot of residence time in days of the tracheal cannula (*x*-axis, independent variable) and length of stay in the ICU in days (*y*-axis, dependent variable) with line of origin, regression line, regression model, and information about goodness of fit.

To demonstrate the interdependence of days with a cannula and LOS in the ICU, we calculated linear regression, with cannulated days in the ICU as the predictor variable for LOS in the ICU. The coefficient of determination showed that 77% (*p* < 0.001) of the variation in LOS can be explained by days with a cannula in the ICU, while the rest is explained by some other variables not included (Figure 9).

**Figure 10:**
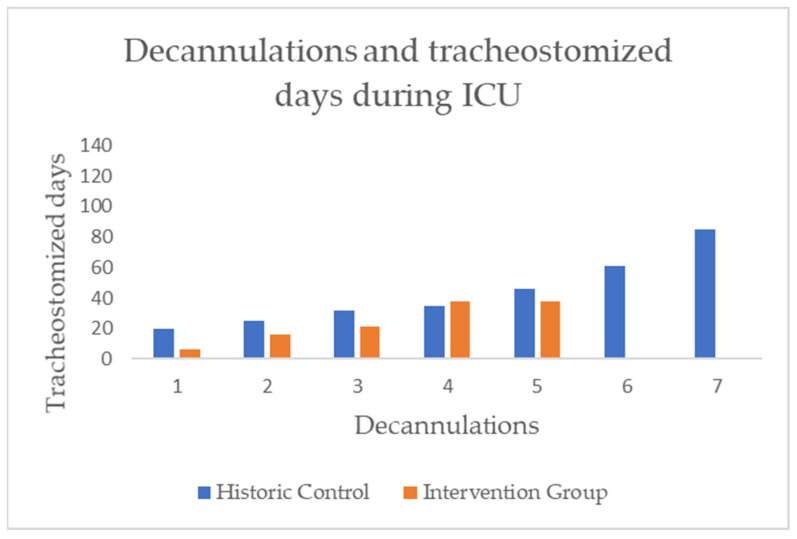
Bar chart of individual tracheostomized days in ICU until decannulation took place, differentiated by group.

In order to look at the effect on SLT and decannulation alone, we analyzed the subgroup of patients who were decannulated during their ICU stay. We therefore used the dates of successful attempts. Regarding the frequency of decannulation, we found no significant difference between the two groups. In the HC, there were seven (12.96%) successfully decannulations, and in the IG, there were five (8.62%) (see Figure 10/Table 2).

**Figure 11:**
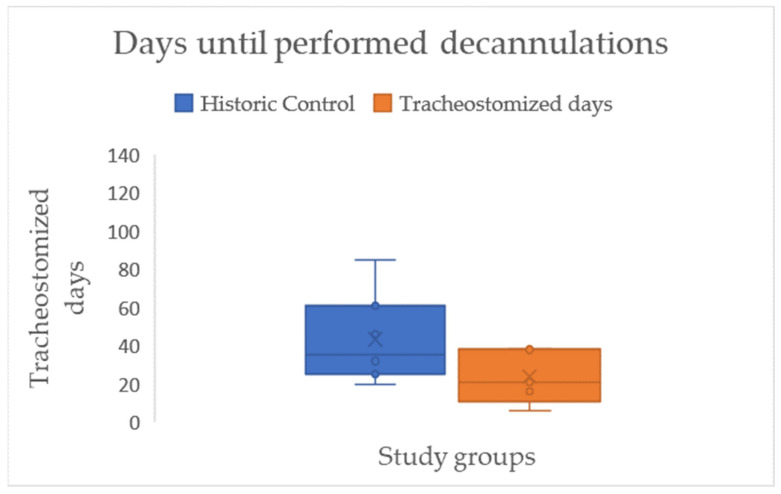
Boxplots with median (-), mean (x), interquartile range (IQR box), whiskers (1.5 × IQR), and outliers (°) for the residence time of cannulas, in days, amongst those who were decannulated during the ICU stay, differentiated by study groups.

In the next step of the analysis, we focused on the residence time of cannulas in those who could be decannulated. A large effect size (d = 0.99), but no statistical significance, was demonstrated for the time difference in days with a cannula between the two groups (HC = 43.43, IG = 23.85) (see Figure 11/Table 2).

**Figure 12:**
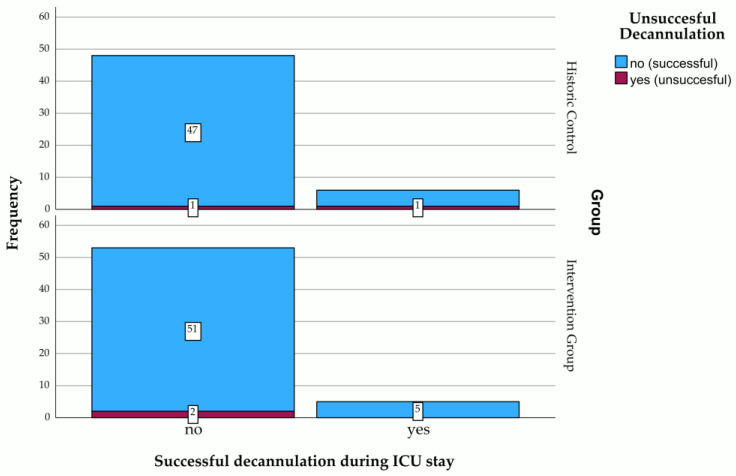
Stacked bar chart of unsuccessful decannulation attempts in those who were either finally decannulated during their ICU stay or not, differentiated by study groups. Figures represent the frequency of each category.

In order to evaluate the developed criteria for assessing readiness for decannulation, and as a safety measure, we counted the number of unsuccessful decannulation attempts and compared the two groups in this respect. In total, there were four unsuccessful decannulation attempts, two in each group. A slightly different distribution was found between the subgroups in which the unsuccessful attempts occurred. Within the IG, where there was a specific protocol to assess readiness for decannulation (see Figure 4), there were no unsuccessful attempts within the subgroup of patients who underwent a final successful decannulation. Unsuccessful attempts occurred only in the subgroup that could not be decannulated during their stay in the ICU. For a full overview, see Figure 12.

The final part of the analysis is related to SLT, its integration into the ICU setting, and its qualitative impact on the project. SLT consisted of individualized cannula management and targeted rehabilitative interventions (see Figure 2 and Figure 3). The allocation of SLT to the dedicated patients was possible for about 65% in the IG, and this was significantly higher and more intensive (d = 0.739) than the only 9% of patients in the HC who received SLT during their ICU stay. This also impacted the diagnostic component of patient care and led to significantly more clinical dysphagia examinations (CDAs), as well as Flexible Endoscopic Evaluations of Swallowing. On the other hand, it did not lead to more patients who were fed by mouth (HC = 11.11% vs. IG = 13.79%) (see Table 2).

Regarding the competence of self-care for their cannula (i.e., self-suctioning), we unfortunately cannot report results, since it was not possible to gather reliable information from the electronic patient documentation systems on this topic.

## 4. Discussion

After implementation of the IQ-ICU project, we were able to demonstrate a mean reduction in days tracheostomized in the ICU by eight days and a reduction in LOS by twelve days for all patients. Concerning those who could be decannulated during their ICU stay, highly clinically relevant results with a large effect size (d = 0.99) could be demonstrated by a mean reduction in the residence time of the tracheal cannula by 20 days. Due to the small sample of performed decannulations (HC *n* = 7, IG *n* = 5), this did not reach statistical significance.

The mean reduction in the residence time of the tracheal cannula is in line with a comparable pre–post study investigating the effect of a tracheostomy pathway [12]. Also, for the direct group comparison of those who could be decannulated in the ICU, the observed difference of 20 days indicates that there are similar effects to those described after the introduction of early rehabilitation concepts (30 days) [23].

Our result that the decannulation process can be sped up but that the number of patients who can be decannulated remains stable has also been shown by others [23]. No more decannulations are possible, which can mean that there might be some “iatrogenic” barrier for possible decannulations that cannot be “bypassed” by traditional means of tracheostomy management (see Section 4.2, Ideas for Future Research).

As demonstrated by the regression model, which exhibited an explained variance of 77%, the days with a cannula can be regarded as a robust predictor of length of stay (LOS). This is noteworthy despite the fact that it functions as a surrogate parameter for a variety of clinical conditions. Hence, it seems very likely that efforts to reduce the residence time of tracheal cannulas will directly translate into reduced LOS in the ICU. This perspective is distinctive in that research has either sought to predict decannulation success [24,25] or LOS [26].

The evaluation of the developed criteria for assessing readiness for decannulation revealed no significant group difference with respect to frequency of unsuccessful attempts to decannulate (HC: 3.7% vs. IG: 3.45%). However, a slightly different distribution was found between the subgroups in which these unsuccessful attempts occurred. Within the IG, there were no unsuccessful attempts in the subgroup of patients who underwent a final successful decannulation during their ICU stay. Due to the limited number of cases (two in each group), it seems inappropriate to interpret these results with respect to the chosen decannulation parameters. With regard to the consequences of this non-finding, please refer to the considerations set out in Section 4.2.

When focusing on the feasibility of integrating SLT in the ICU setting, we were able to allocate SLT to 65% of dedicated patients in the IG. This resulted in significantly more treatment (d = 0.739) and also impacted the diagnostic component of patient care. with significantly more clinical dysphagia examinations (CDAs), as well as Flexible Endoscopic Evaluations of Swallowing. Hence, we conclude that this is evidence for the practical applicability of respective guideline recommendations [6,7]. On the other hand, this did not lead to more patients who were fed by mouth (HC = 11.11% vs. IG = 13.79%), although this cannot be considered a primary goal for ICUs and can always be addressed in subsequent normal wards.

### 4.1. Limitations

First of all, limitations arise from the pre–post study design. A randomized controlled trial would have been a more desirable study design, but it was not feasible to implement two different projects in the same ICU ward with the same staff simultaneously. Also the retrospective data analysis for the HC bears risks of bias. For instance, a strong source of bias would have been data from the COVID-19 period. Consequently, the retrospective analysis was constrained to the post-COVID-19 period, with sufficient temporal distance.

The fact that no more decannulations were possible in the IG than in the HC, as we had expected, could have been influenced by the differences in the tracheostomy types between the two groups. There were significantly more surgical tracheostomies in the IG (12) than in the HC, contributing to more efforts for decannulation (e.g., the necessary surgical closure of the stoma) [27]. A potential source of bias, contributing to a shorter LOS in the IG, and hence a reverse effect to the one previously discussed, could be the mean difference in time of tracheostomy (first day with a TC in the ICU) of four days between the two groups (HC = 13.93 → IG = 9.7). This is because there is relevant evidence that found early tracheostomy to result in fewer ICU days [28]. However, given that both groups can be classified as “early tracheostomies” and that we identified both an inhibiting and a promoting potential bias within the same group (i.e., our IG), it is anticipated that these biases will offset each other.

### 4.2. Considerations and Ideas for Future Research

For a better understanding of the effect of SLT on the reduction in time until decannulation in the IG, we will perform a detailed case study of these patients as a next step.

In order to keep up with state-of-the-art research, we want to implement the Standardized Endoscopic Swallowing Evaluation for Tracheostomy Decannulation (SESETD) protocol [29] in the IQ-ICU-SLT SOP.

As explained, due to uncertainty regarding whether ICU patients would meet the necessary etiologies for PES application (i.e., neurogenic dysphagia in Europe), we decided against PES at the first stage of the IQ-ICU project. Nevertheless, its proven usefulness in speeding up the decannulation process [13] makes it an interesting tool. Since across both of our groups, the complication of apoplexy emerged in 15.18%, with an associated high risk of neurogenic and post-extubation dysphagia [30], we consider PES a potential future means of PED prevention. Hence, PES could be integrated into the weaning from ventilation process during the early intubation phase in order to reduce PED and extubation failure/or necessary tracheostomies, with the potential to further shorten the LOS in the ICU. Therefore, includable etiologies (e.g., stroke, brain tumor, traumatic brain injury) or clinical symptoms indicating neurogenic dysphagia (e.g., tolerance of endotracheal tubes, saliva pooling) that potentially would benefit from PES application in general ICU wards have to be identified. By this, the PED could be reduced, and this might result in either a lower necessity for tracheostomy overall or more decannulations that can be immediately performed in the ICU and not later in course of rehabilitation.

The demonstrated feasibility of implementing SLT in tracheostomy management in an ICU and the accompanying positive results of IQ-ICU-SLT should be acknowledged by professional societies and associations and lead to clear recommendations for the inclusion of SLT in ICUs, as already demonstrated in one guideline [7].

## 5. Conclusions

This project demonstrated that interprofessional teamwork between medicine, nursing, physiotherapy, SLT, and occupational therapy in a general ICU is feasible and can improve the quality of treatment and reduce morbidity, as well as LOS in the ICU. Integrating SLT and its expertise into intensive care will likely lead to benefits such as shorter times to decannulation and safer decannulation processes, since necessary diagnostics like FEES can be performed more often and by specially trained staff. This also enhances patients’ quality of life.

Surely, the demonstrated positive effects on the reduction in LOS cannot be solely attributed to SLT input on treatment. However, the demonstrated reduction in days until possible and safe decannulation can be considered an indicator of the effective integration of SLT into this process in general ICUs.

## Figures and Tables

**Table 1:** Patient characteristics (*n* = 112) with means, standard deviations (SDs), and frequencies during the ICU stay for both groups (historic control, HC, and intervention group, IG,) with analysis of potential differences in distribution.

	HC (*n* = 54)	IG (*n* = 58)	Difference	Significance
General Characteristics				
Age	Mean = 65.43 SD = 13.15	Mean = 67.75 SD = 13.56	Not sig. unequal	*t*-test: *p* = 0.361
Sex (F/M)	F = 29.6% M = 70.4%	F = 44.8% M = 55.2%	F < M in HC	X^2^: *p* = 0.003 *
BMI	Mean = 27.0 SD = 4.97	Mean = 28.06 SD = 9.21	Not sig. unequal	*t*-test: *p* = 0.361
Morbidity/Mortality				
SAPS-2 max ^1^	Mean = 80.22 SD = 13.86	Mean = 80.86 SD = 14.98	Not sig. unequal	*t*-test: *p* = 0.822
SOFA max ^2^	Mean = 15.08 SD = 2.85	Mean = 13.78 SD = 4.35	HC > IG	*t*-test: *p* = 0.004 *
Clinical Frailty Scale ^3^	Mean = 4.04 SD = 1.66	Mean = 3.72 SD = 1.36	Not sig. unequal	*t*-test: *p* = 0.278
Complication Apoplexy (%)	11 (20.4%)	6 (10.3%)	Not sig. unequal	X^2^: *p* = 0.140
Death on ICU (%)	10 (18.5%)	4 (5.9%)	Not sig. unequal	X^2^: *p* = 0.063
Tracheostomy				
Types ^4^ (Frequency)	Prim. surg. = 1 Second. surg. = 0 Perc. = 53	Prim. surg. = 12 Second. surg. = 7 Perc. = 39	Prim. and second. surgical: IG > HC	X^2^: *p* < 0.001 *
First day with tracheostomy on ICU	Mean = 13.44 SD = 10.17	Mean = 9.71 SD = 8.02	IG earlier than HC	*t*-test: *p* = 0.027 *

^1^ SAPS II, 0–163, risk of mortality; ^2^ SOFA, 0–24, organ dysfunction; ^3^ CFS, 0–9, frailty; ^4^ types of tracheostomies: primary surgical, secondary surgical, and percutaneous; * denotes statistical significance (*p* ≤ 0.05).

**Table 2:** Primary SLT-specific (P1) and general (P2) results, as well as secondary outcomes, between the historic control (HC) and intervention group (IG) with differences, statistical significance (* denotes *p* ≤ 0.05), and effect sizes (ESs) for the most relevant outcomes.

	HC (*n* = 54)	IG (*n* = 58)	Difference	Significance/Effect Size (d)
Days with cannula in ICU (P1)	Mean = 18.85 SD = 19.29	Mean = 10.31 SD = 12.1	IG < HC	*t*-test: *p* = 0.006 * d = 0.54
LOS-ICU (P2)	Mean = 33.74 SD = 26.73	Mean = 21.91 SD = 22.49	IG < HC	*t*-test: *p* = 0.012 * d = 0.48
Successful decannulations (%)	7 (12.96%)	5 (8.62%)	Not sig. unequal	X^2^: *p* = 0.658
Unsuccessful decannulations (%)	2 (3.7%)	2 (3.45%)	Not sig. unequal	X^2^: *p* = 0.942
Days until successful decannulation	Mean = 43.43 SD = 22.85	Mean = 23.85 SD = 14.04	IG earlier HC	*t*-test: *p* = 0.121 d = 0.99
FEES per Patient	Mean = 0.018 SD = 0.13	Mean = 0.14 SD = 0.44	IG > HC	*t*-test: *p* = 0.007 *
Clinical Dysphagia Examinations (%)	7 (12.96%)	26 (44.83%)	IG > HC	X^2^: *p* < 0.001 *
Patients fed by mouth (%)	6 (11.11%)	8 (13.79%)	Not sig. unequal	X^2^: *p* = 0.668
Patients with SLT (%)	5 (9.26%)	38 (65.52%)	IG > HC	X^2^: *p* < 0.001 *
SLT minutes in ICU	Mean = 23.33 SD = 91.22	Mean = 358.45 SD = 623.56	IG > HC	*t*-test: *p* < 0.001 * d = 0.739

* denotes statistical significance (*p* ≤ 0.05).

## Data Availability

The data are not publicly available due to restrictions on the use of clinical patient data. Furthermore, a public accessibility is not covered by the given ethics vote. In this, data sharing is limited to inner institutional research partners.
